# Comparative diagnostic efficacy of swab stick and pipette sampling techniques for vaginal cytology in oestrous detection of West African Dwarf Goats

**DOI:** 10.3389/fvets.2026.1768722

**Published:** 2026-05-04

**Authors:** Ugochinyere J. Njoga, Ugochi M. Nwaibe, Ibe N. Patrick, Onoh E. Chukwubuike, Izuchukwu S. Ochiogu, John I. Ihedioha, Kenneth O. Anya, Emmanuel O. Njoga, James W. Oguttu

**Affiliations:** 1 Department of Veterinary Obstetrics and Reproductive Diseases, Faculty of Veterinary Medicine, University of Nigeria, Nsukka, Nigeria; 2 Department of Veterinary Pathology, Faculty of Veterinary Medicine, University of Nigeria, Nsukka, Nigeria; 3 Department of Agriculture and Animal Health, College of Agriculture Environmental Sciences, University of South Africa, Florida Campus, Johannesburg, South Africa; 4 Department of Public Health and Preventive Medicine, Faculty of Veterinary Medicine, University of Nigeria, Nsukka, Nigeria

**Keywords:** debris, diagnostic cost-effectiveness, oestrous cycle, pipette method, smear quality, swab stick method, vaginal cytology, West African Dwarf goats

## Abstract

**Background:**

Accurate detection and monitoring of the oestrous cycle in West African Dwarf (WAD) goats is indispensable for reproductive management. Vaginal cytology is commonly used, but different collection methods may vary in diagnostic efficacy, smear quality, and animal safety. This study compared the diagnostic efficacy of pipette and swab stick methods for monitoring the oestrous cycle of WAD goats.

**Methods:**

Four cyclic WAD goats were sampled repeatedly for four consecutive days using both pipette and swab stick methods. A total of 16 samples were collected from each doe during the 4-day sampling period. Smears were evaluated for cell types (parabasal, intermediate, superficial), debris, and cellular distortion. Animal response to sampling was assessed via bleating and presence of blood cells to determine trauma. Economic and comprehensive diagnostic cost-effectiveness were also evaluated. Data were analyzed using paired-sample *t*-test, Chi-square, and Fisher’s Exact Test where appropriate.

**Results:**

Swab stick smears yielded a higher proportion of superficial cells (9.41 ± 2.35) than pipette smears (4.04 ± 0.93, *p* = 0.03), while parabasal cells were higher in pipette smears (1.97 ± 0.43 vs. 0.28 ± 0.08, *p* = 0.001). Heavy debris was observed more frequently in pipette smears (68.8%) than swab stick smears (25.0%, *p* = 0.032). No pipette smears exhibited cellular distortion, whereas swab stick smears showed mild to severe distortion (Fisher’s Exact Test, *p* = 0.007). Pipette sample collection caused greater trauma, indicated by prolonged bleating and moderate blood cell presence, compared with swab sampling. Although pipettes were more economical due to their lower cost and reusability, swab sticks demonstrated higher overall diagnostic cost-effectiveness when factors such as smear quality, trauma, and diagnostic yield were taken into account.

**Conclusion:**

Both methods are effective for monitoring the oestrous cycle in WAD goats; however, swab sticks offer higher diagnostic yield in detecting heat and lower trauma, whereas pipettes are more economical. Selection of method should balance cost, diagnostic performance, and animal welfare. Future studies with larger sample sizes are recommended to confirm these findings.

## Introduction

Small ruminant production is an important component of livestock agriculture in many developing countries, particularly in Sub-Saharan Africa, where it contributes significantly to household nutrition, income generation, display of affluence in rural regions and poverty alleviation (1, 2). In Nigeria, goats represent the second-largest livestock population after poultry, with an estimated 76 million goats, of which the West African Dwarf (WAD) goat constitutes the dominant indigenous breed (3). These goats are highly adaptable to diverse agricultural zones, tolerant to some endemic diseases like trypanosomiasis (4, 5), and valued for their short gestation interval, twinning rates, and meat and milk production potentials (6, 7). Despite these advantages, productivity in smallholder goat systems remains below potential, largely due to reproductive inefficiencies and poor husbandry management (6).

Reproductive management is fundamental to optimizing goat production (8). Accurate detection of oestrus is vital for efficient mating, artificial insemination, oestrus synchronization success and genetic improvement programs (9, 10). Oestrus detection in goats is often challenging because behavioral signs, such as vulvar swelling, tail wagging, restlessness, and vocalization, may be subtle or absent, particularly under intensive management systems due to short duration of oestrus (24 h) (11, 12). Reliance on male teaser goats for heat detection is common (13, 14), but this approach is labor-intensive, less precise, and may increase disease transmission risks. These challenges underscore the need for objective and practical diagnostic tools to monitor the oestrous cycle.

Exfoliative vaginal cytology has been recognized as a reliable, inexpensive, and non-invasive technique for assessing oestrous cycle stages in domestic animals (15). The method is based on changes in the proportion of parabasal and intermediate (non-cornified), superficial (cornified) epithelial cells and leucocytes in vaginal smears, reflecting fluctuating steroid hormone levels throughout the oestrous cycle (16). During proestrus and oestrus, superficial cells predominate under estrogenic stimulation, whereas dioestrus is characterized by higher proportions of parabasal and intermediate cells under progesterone influence (11, 17, 18). The utility of vaginal cytology for oestrus detection and pregnancy diagnosis has been well documented in small ruminants (11, 19).

Several techniques have been described for the collection of vaginal cytology samples in goats, including the use of cotton swabs, glass slides, spatulas, and pipettes methods (11, 13, 19–23). Sample collection and preparation techniques may significantly influence smear quality and diagnostic accuracy and sample quality (24). The swab stick method is most commonly employed approach due to its simplicity and ability to yield clean smears (15). Conversely, the pipette method is cost-effective and reusable (20) making it attractive for both field and laboratory applications; However, it may produce smears with increased debris and background material, potentially compromising cytological interpretation. Such methodological differences may directly affect the accuracy and reliability of oestrous cycle determination.

Despite the extensive application of exfoliative vaginal cytology in small ruminants, systematic comparisons of sampling techniques in goats remain limited, particularly in indigenous breeds such as the West African Dwarf (WAD) goat. Previous studies have mainly focused on describing cytological patterns across the oestrous cycle (15, 16). However, comparative evaluations of commonly used sampling methods- specifically swab stick versus pipette techniques- regarding diagnostic efficacy, animal welfare, and cost-effectiveness are scarce. Bridging this knowledge gap is essential to optimize cytological protocols for reliable oestrous detection under both field and controlled management conditions.

It was therefore hypothesized that the swab stick method would provide superior diagnostic efficacy for oestrus detection with minimal vaginal trauma compared with the pipette method, while the pipette method would be more cost-effective. Accordingly, the present study aimed to compare the swab stick and pipette methods of vaginal cytology in WAD goats with emphasis on three key parameters: (i) diagnostic efficacy based on epithelial cell proportions, (ii) trauma evaluation based on animal discomfort and presence of blood cells, and (iii) cost-effectiveness in relation to field and laboratory applications. By addressing this gap, the study provides evidence-based guidance for decision making regarding the selection of appropriate cytological techniques, while balancing diagnostic efficacy and animal welfare. In the long term, these findings are expected to contribute to improved reproductive efficiency and the sustainability of small ruminant production systems, with broader implications for food security.

## Methodology

### Study location

The study was conducted at the Small Ruminant Research Unit, Department of Veterinary Obstetrics and Reproductive Diseases, Faculty of Veterinary Medicine, University of Nigeria, Nsukka, South East Nigeria. The zone has been described previously by Njoga et al. (25) to be located at latitude 5^°^45^0^00^00^ N and longitude 8^°^30^0^00^00^ E. The climate of the study area is characterized by a humid tropical environment with a mean annual rainfall of approximately 1700 mm and mean ambient temperature of 27–30 °C.

### Experimental animals and management

**Figure 1:**
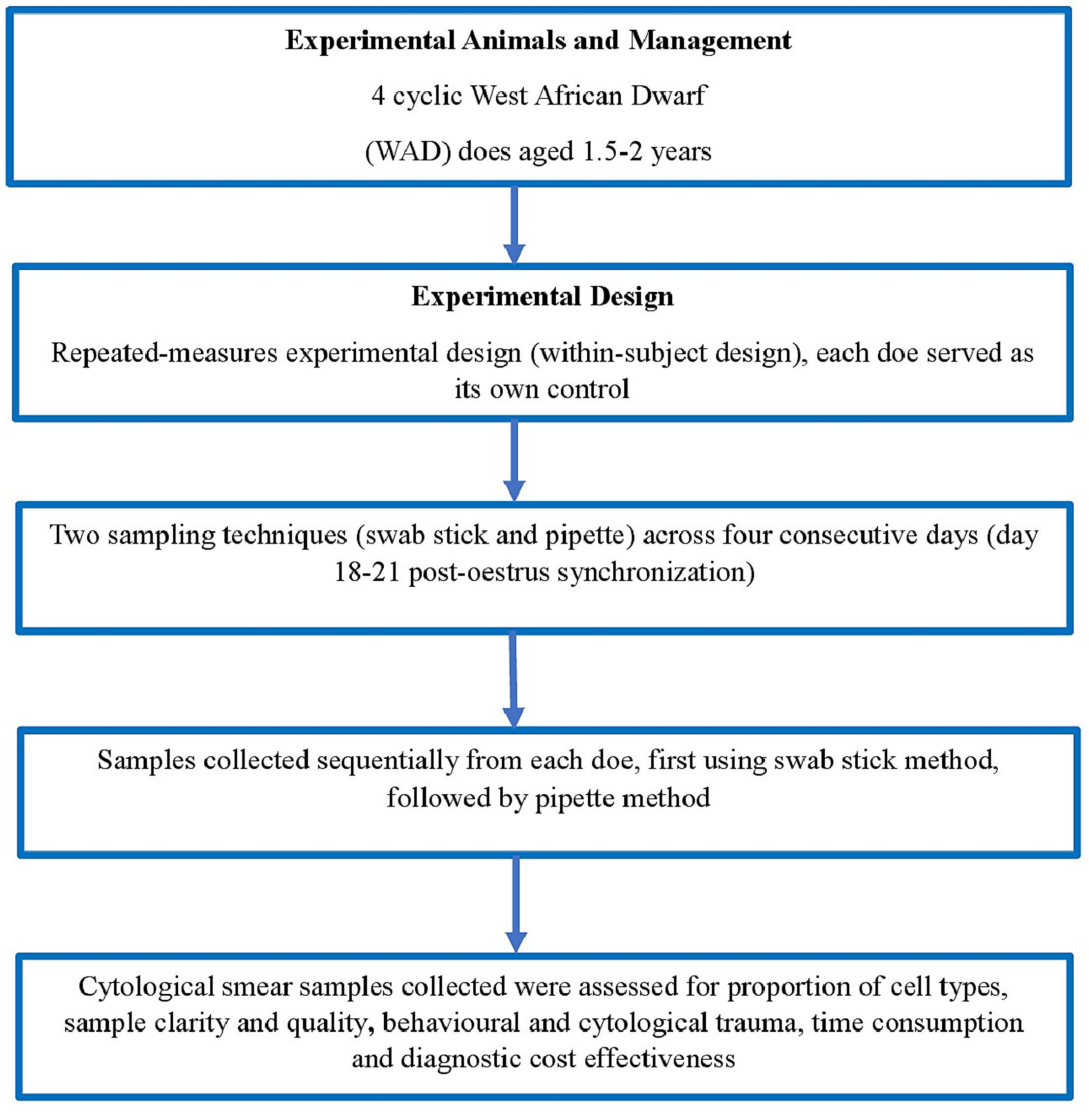
Experimental design and workflow of the comparative evaluation of swab stick and pipette methods in WAD does.

A total of 4 cyclic West African Dwarf (WAD) does aged 1.5–2 years and weighing 10–12 kg was used for this study (Figure 1). Only clinically healthy animals with a history of regular oestrous cycles were included. Goats were maintained under a semi-intensive management system, allowed daily access to natural grazing, and supplemented with bambaranut cake chaff and clean drinking water ad libitum. Deworming and prophylactic vaccinations (pesti des petit ruminanti) were carried out before the commencement of the experiment.

### Experimental design

This study employed a repeated-measures experimental design (within-subject design) in which each doe served as its own control and a biological replicate (Figure 1). This approach increases statistical efficiency and minimizes animal use, consistent with ethical guidelines. All does were subjected to two vaginal cytology sampling techniques (swab stick and pipette) across four consecutive days, beginning on day 18 (corresponding to the onset of the follicular phase in a 17-day oestrous cycle) and continuing until day 21 post-oestrus synchronization. Sampling was performed by one operator at a defined stage corresponding to proestrus based, on cycle monitoring following synchronization protocol. Samples were collected sequentially from each doe, first using the swab stick method followed by the pipette method. A total of 16 samples were collected per doe during the 4-day sampling period. Cytological smear samples collected were evaluated for epithelial cell proportions, sample clarity and quality, Indicators of behavioral and cytological trauma, time requirements and diagnostic cost- effectiveness as presented in Figure 1. All animals were humanely handled in accordance with the ethical guidelines of the Institutional Animal Care and Use Committee (IACUC), Faculty of Veterinary Medicine, University of Nigeria, Nsukka. Ethical approval for the study was granted under approval number FVM-UNN-IACUC-2025-06/245.

### Synchronization protocol

A progesterone-based estrus synchronization protocol using a Controlled Internal Drug Release (CIDR^®^) implant (EAZI-BREED™ CIDR^®^ Sheep insert, containing 0.3 g of progesterone in molded silicone over a flexible nylon spine), in combination with dinoprost tromethamine injection (5 mg/mL), a luteolytic agent, was employed for estrus synchronization as described by Lv et al. (26), with minor modifications. The CIDR^®^ implant was inserted into the vagina using an obstetrical applicator and left *in situ* for 9 days, rather than 7 days as described in the reference protocol, to mimic the luteal phase. Additionally, to induce luteolysis, 2 mL of dinoprost tromethamine (5 mg/mL) was administered intramuscularly to each goat, 24 h prior to CIDR^®^ removal (day 8), instead of at the time of removal. Exogenous gonadotropins (eCG) and GnRH injections used in the reference study were not included, as the objective was to evaluate vaginal cytology techniques under a simplified, field-applicable synchronization protocol.

### Swab stick method

The swab stick method was performed following procedures previously described by Cowell and Tyler (22) and OECD (20). The vulva of each goat was cleaned with an antiseptic solution (chlorhexidine gluconate) prior to daily sample collection. A sterile cotton-tipped swab stick, pre-moistened with normal saline (9%), was gently inserted approximately 5 cm into the vaginal canal in a cranio-dorsal direction and rotated against the mucosal wall to collect exfoliated vaginal epithelial cells. The swab was then rolled evenly onto a clean glass slide to prepare a thin, uniform smear. The slide was air-dried and fixed in 100% methanol for 2 min, then rinsed with clean tap water. Finally, the slide was stained with Giemsa stain, diluted 1:10in buffered distilled water. The stain was allowed to act for 30 min, after which the slides were rinsed and examined under a light microscope at ×40 magnification.

### Pipette method

The pipette method was conducted in accordance with the guidelines described by OECD (20), Cora et al. (21), and Nishvanth et al. (23). A sterile Pasteur pipette fitted with a rubber bulb, containing 1 mL of normal saline (0.9%), was inserted into the vaginal canal. The normal saline was released into the vaginal canal and vaginal secretions containing epithelial cells were subsequently aspirated. This procedure was repeated twice, after which a drop of the aspirated fluid was placed onto a clean glass slide. The slide was kept in a slanted position to allow the drop run along the entire length of the slide, forming a thin smear. The smears were air-dried, fixed and stained as described above. Epithelial cells were classified into three morphological categories: parabasal, intermediate, and superficial cells, as described by Siregar et al. (17).

### Diagnostic efficacy of swab stick and pipette sampling methods

The diagnostic efficacy of the two methods was assessed based on cellular yield specifically the proportions of parabasal, intermediate and superficial epithelial cells obtained from each method. Vaginal smears were examined and cells were counted under a light microscope at ×40 magnification. All observable epithelial cells within each smear were counted, and the relative percentage of each cell type was calculated. A collection technique was considered more diagnostically effective for oestrus detection if it yielded a higher proportion of superficial cells, which are indicative of the oestrus phase.

### Trauma evaluation

The degree of trauma induced by each sampling method was evaluated using a scoring system that incorporated behavioral and cytological smear parameters:

Bleating response: Pain-related vocalization was scored as – (absent, <3 s) or + (present, >3 s), adapted from Luginbuhl (13), who reported that does may vocalize loudly during stressful or painful events, including handling during the oestrous period.
Blood on smears: Cytological trauma was evaluated based on the presence of blood cells on vaginal smears, graded as none (no blood cells), mild (few scattered blood cells per field), moderate (multiple blood cells per microscopic field), or severe (confluent blood cells obscuring cytological evaluation), following Cowell and Tyler (22). Each parameter (bleating response and presence of blood cells) was evaluated independently, and the overall trauma assessment was based on the combined interpretation of these parameters, with the pipette and swab stick methods classified as relatively high or low. This approach was chosen because behavioral responses and blood contamination are widely recognized as reliable indicators of procedural trauma in veterinary cytology.

### Cytological clarity and sample quality

Two parameters were used to evaluate the diagnostic quality of vaginal smears: Cellular distortion: Cellular distortion was assessed as the proportion of vaginal epithelial cells exhibiting morphological distortion per microscopic slide, based on overall slide evaluation. Smears were classified as *none* (0% distorted cells), *mild* (<10%), *moderate* (10–30%), or *severe* (>30% distorted cells). Each vaginal smear was assigned a single distortion category based on the estimated proportion of distorted cells observed across the entire slide. For overall diagnostic cost-effectiveness, cellular distortion was dichotomized as *present* (mild–severe) or *absent* (none) to ensure methodological consistency. Background debris: The presence of cellular debris in vaginal smears was graded as none, mild, moderate, or heavy based on the overall density of non-cellular material observed per microscopic slide. However, for inferential statistical analysis, these categories were collapsed into two groups: “Heavy”, representing smears with dense debris likely to interfere with cytological interpretation, and “Not Heavy”, which combined None, Mild, and Moderate debris. This categorization was applied to enhance statistical robustness by ensuring adequate expected cell frequencies for Pearson’s Chi-square and Fisher’s Exact tests, while retaining the diagnostic relevance of debris severity.

### Unit cost and operational characteristics of sampling methods

Cost analysis was conducted by recording the unit market prices of swab sticks and pipettes at the time of the study. In addition, the reusability of pipettes and the single-use nature of swab sticks were documented. Overall unit cost was determined based on the combined consideration of unit price and reusability, with collection methods qualitatively classified as “high” or “low”. This approach was adopted to reflect practical field and laboratory conditions commonly encountered in small ruminant reproductive studies.

### Diagnostic cost-effectiveness

The diagnostic cost-effectiveness of the two sampling methods was evaluated by integrating smear quality (cellular distortion and background debris), extent of trauma, and diagnostic yield, defined as the proportion of superficial epithelial cells obtained. Overall diagnostic cost-effectiveness of each method was qualitatively rated as either “Moderate” or “High.”

### Assessment of time efficiency

The time required to complete each sampling method was measured in seconds using a digital mobile phone stopwatch. Timing commenced at the point of vaginal sample collection and ended once a smear was prepared on a clean glass slide. This measurement was repeated for each sample collection to determine the relative time efficiency of the two methods during each sampling event.

### Statistical analysis

Data were analyzed using SPSS software (version 21.0; IBM Corp., Armonk, NY, United States). Continuous variables (proportions of epithelial cell types, time consumption) were expressed as mean ± SEM. Normality of the difference scores was assessed using the Shapiro–Wilk test and was not significant (0.091), indicating that the data were normally distributed. Therefore, paired-sample *t*-tests were used to compare pipette and swab stick methods, using the mean per animal across 4 consecutive sampling days. Categorical variables (cellular distortion, debris) were analyzed using Chi-square or Fisher’s Exact test when expected counts were <5. Trauma parameters and cost-effectiveness ratings were presented descriptively due to small sample size and qualitative nature. All statistical tests were two-tailed, and *p*-values ≤0.05 were considered statistically significant.

## Result

### Diagnostic efficacy of swab stick and pipette sampling methods

The diagnostic performance of the two sampling techniques is summarized in Table 1. Parabasal cells were significantly more abundant (t = 4.06, *p* = 0.001, Cohen’s d = 1.01) in samples collected using the pipette method (1.97 ± 0.43) compared with the swab stick method (0.28 ± 0.08). Conversely, the proportion of superficial cells was significantly higher (t = −2.34, *p* = 0.034, Cohen’s d = 0.58) in samples obtained using the swab stick method (9.41 ± 2.35) than with the pipette method (4.04 ± 0.93). The proportion of intermediate cells did not differ significantly (t = −0.497, *p* = 0.626, Cohen’s d = 0.12) between the pipette (86.85 ± 5.63) and swab stick (90.15 ± 2.36) methods.

**Table 1:** Comparison of epithelial cell-type proportions in West African Dwarf (WAD) goat vaginal smears collected using pipette and swab methods.

Cell type	Pipette (mean ± SEM)	Swab (mean ± SEM)	*p*-value
Parabasal	1.97 ± 0.43	0.28 ± 0.08	0.001*
Intermediate	86.85 ± 5.63	90.15 ± 2.36	0.620
Superficial	4.04 ± 0.93	9.41 ± 2.35	0.030*

Data are presented as Mean ± SEM. Comparisons were performed using paired-samples *t*-tests. * Indicates statistical significance sat *p* ≤ 0.05.

### Cytological clarity and sample quality

#### Cellular distortion

**Figure 2:**
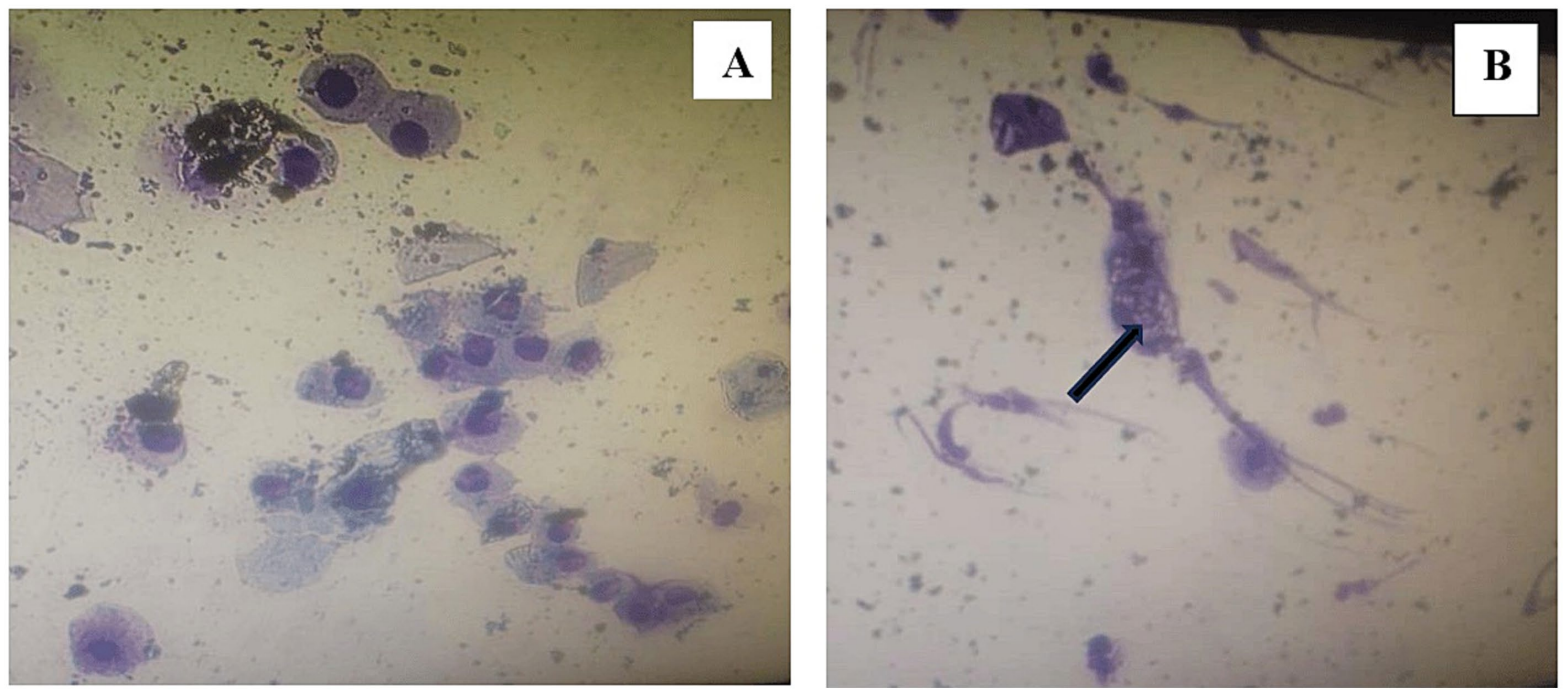
Comparison of cellular distortion between sampling methods, **(A)** pipette method; **(B)** swab stick method. The black arrow indicates cellular distortion observed in the swab stick smears.

The degree of cellular distortion differed significantly between collection methods (Table 2). All pipette smears (100%; Figure 2A) showed no distortion, whereas the swab smears displayed mild (25.0%), moderate (12.5%), and severe (6.3%) distortion (Figure 2B). Due to low expected counts in some categories, Fisher’s Exact Test was applied, confirming a significant association between collection method and cellular distortion (exact *p* = 0.007).

**Table 2:** Association between sampling method and cellular distortion per vaginal smear in West African Dwarf (WAD) goat vaginal smears.

Method	None (*n*, %)	Mild (*n*, %)	Moderate (*n*, %)	Severe (*n*, %)	Total
Pipette	16 (100.0)	0 (0.0)	0 (0.0)	0 (0.0)	16 (100.0)
Swab	9 (56.3)	4 (25.0)	2 (12.5)	1 (6.3)	16 (100.0)
Total	25 (78.1)	4 (12.5)	2 (6.3)	1 (3.1)	32 (100.0)

Values are presented as counts with percentages in parentheses; each value represents one vaginal smear (one score per microscopic slide). Cellular distortion was scored as *none* (0% distorted cells), *mild* (<10%), *moderate* (10–30%), or *severe* (>30%), based on overall slide assessment. Statistical significance was evaluated using Fisher’s exact test, as more than 75% of the contingency table cells had expected counts <5.

#### Cellular debris

**Figure 3:**
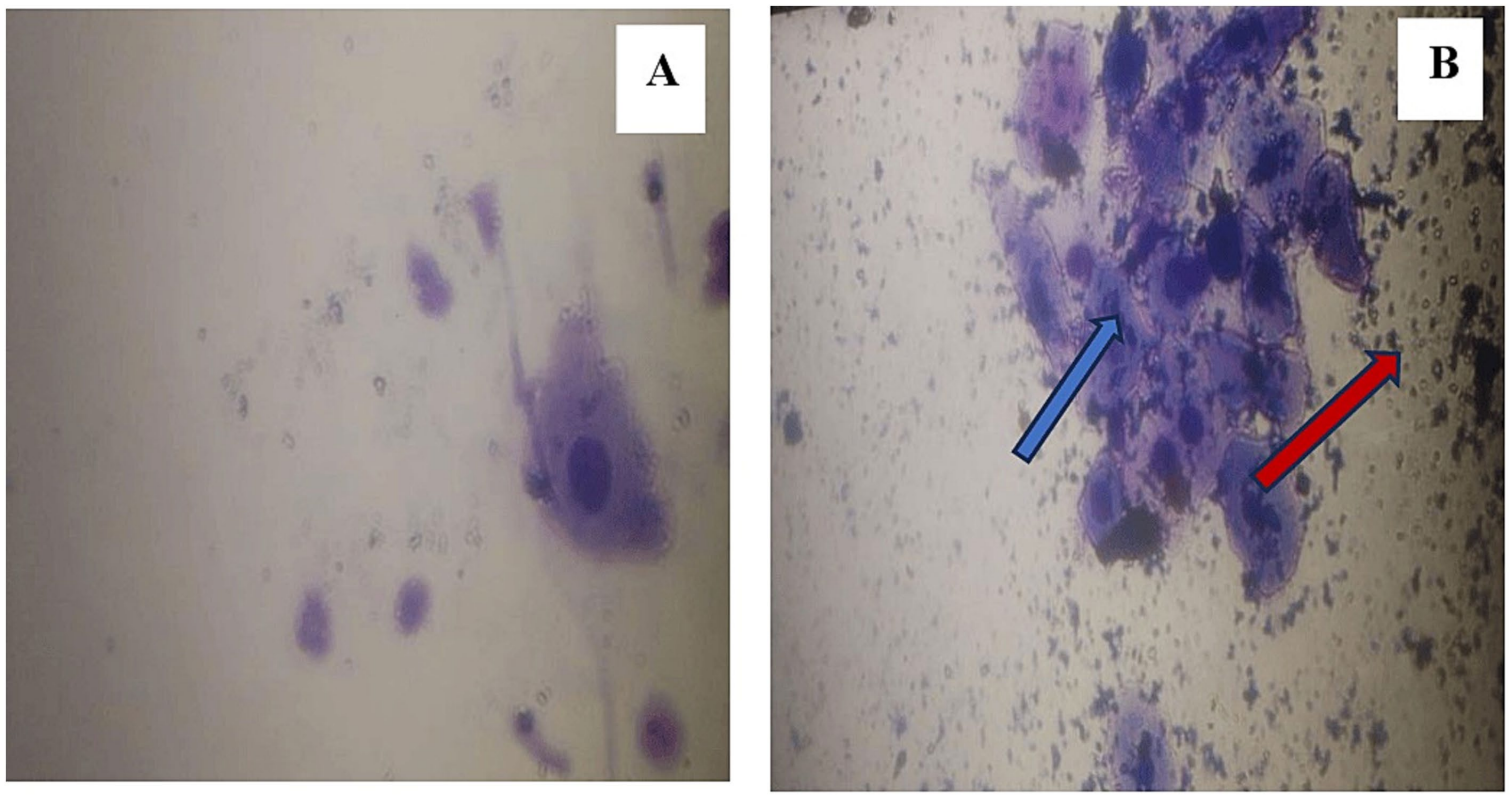
Comparison of debris presence in swab stick **(A)** and pipette **(B)** methods. Heavy cytoplasmic debris in pipette smears (red arrow); debris interfering with epithelial cell identification (blue arrow).

Background debris was classified as “Heavy” or “Not Heavy” (Table 3). Heavy debris occurred more frequently in pipette smears (68.8%; Figure 3B) than in swab stick smears (25.0%; Figure 3A). Pearson Chi-square analysis confirmed a significant association between method and debris category (χ^2^ = 6.149, *p* = 0.013).

**Table 3:** Comparison of cellular debris levels per vaginal smear (slide) in samples collected using pipette and swab stick methods.

Method	Not heavy	Heavy	Total
Pipette	5 (31.3%)	11 (68.8%)	16
Swab	12 (75.0%)	4 (25.0%)	16
Total	17 (53.1%)	15 (46.9%)	32

Each value represents the number of vaginal smears (one score per microscopic slide), with percentages in parentheses. Background debris was assessed across the entire slide and classified as heavy or not heavy. Statistical significance was evaluated using Pearson Chi-square.

### Evaluation of trauma associated with sampling techniques

Trauma was assessed descriptively using behavioral (bleating) and cytological (blood on smears) indicators (Table 4). Bleating >3 s was observed in goats sampled using the pipette method, whereas no prolonged bleating was recorded during swab stick sampling. The presence of blood cells was graded as moderate in pipette-collected smears and mild in smears obtained using the swab stick method. The overall trauma was therefore rated as “high” for the pipette method and “low” for the swab sticks method. These evaluations are descriptive due to the small sample size (*n* = 4).

**Table 4:** Trauma associated with pipette and swab stick methods.

Parameter	Pipette	Swab stick
Bleating response	+	–
Presence of blood cells	Moderate	Mild
Overall trauma	High	Low

“+” Indicates bleating lasting >3 s; “–” indicates bleating lasting <3 s. Mild = few scattered blood cells per field; moderate = multiple blood cells per field.

**Figure 4:**
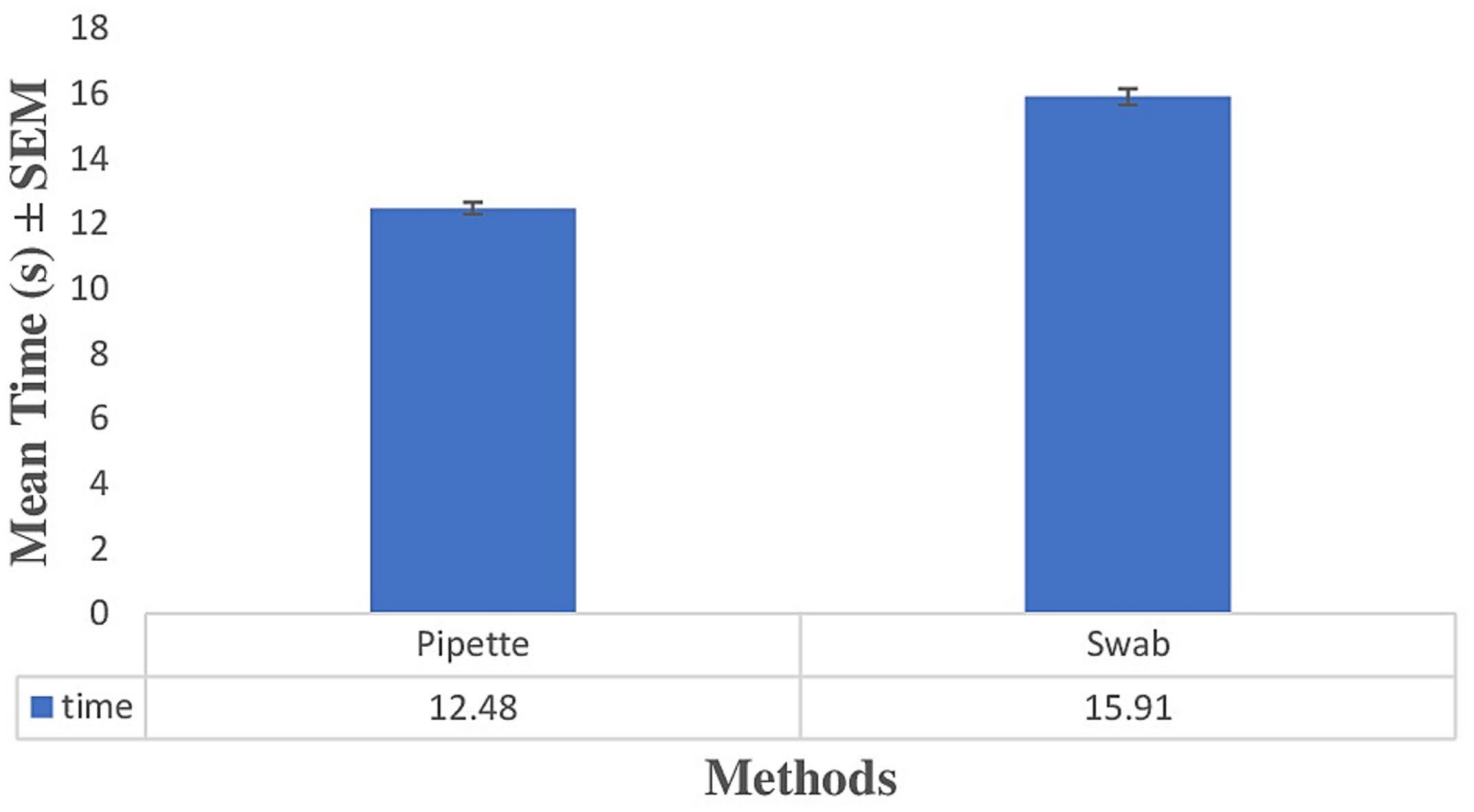
Mean time measurement for pipette and swab stick methods. Error bars represent SEM. Paired *t*-test: *p* < 0.001.

### Unit cost, operational characteristics, and overall diagnostic cost-effectiveness of sampling methods

Economic analysis revealed that pipettes were less costly (₦50, reusable) compared to swab sticks (₦100 per unit, single-use), making pipettes more cost-effective from a price perspective (Table 5). However, when diagnostic parameters including debris, distortion, trauma, and superficial cell yield were considered, swab sticks smears were reported to have higher overall diagnostic cost-effectiveness (Table 6). Therefore, overall diagnostic cost-effectiveness was rated as “Moderate” for the pipette method and “High” for the swab stick method.

**Table 5:** Evaluation of unit cost and operational characteristics of sampling methods.

Cost parameter	Pipette	Swab stick
Price (₦)	50	100
Reusability	✓	×
Unit cost	Low	High

✓ = reusable; × = single-use (non-reusable).

**Table 6:** Overall diagnostic cost-effectiveness of sampling methods.

Parameter	Pipette	Swab stick
Price (₦)	50	100
Reusability	✓	×
Debris	Heavy	Not heavy
Distortion	Absent	Present
Trauma	High	Low
Diagnostic yield (superficial %)	Low	High
Overall diagnostic cost-effectiveness	Moderate	High

✓ = reusable; × = single-use (non-reusable).

### Evaluation of time consumption of swab stick and pipette methods

The mean time required to complete the vaginal cytology procedure using the pipette method (12.48 ± 0.78 min) was significantly shorter than that required for the swab method (15.91 ± 0.98 min). Paired-samples *t*-test analysis revealed a mean difference of −3.43 ± 0.99 min (95% CI = −3.96 to −2.90), which was statistically significant (t (15) = −13.87, *p* < 0.001) (Figure 4).

## Discussion

This study provides a structured comparative evaluation of two commonly used vaginal cytology sampling techniques, swab stick and pipette methods, for oestrous cycle monitoring in West African Dwarf (WAD) goats. While previous studies have demonstrated the usefulness of exfoliative vaginal cytology for identifying oestrous stages in small ruminants, direct methodological comparisons that simultaneously consider diagnostic yield, smear quality, trauma, time efficiency, and cost-effectiveness are limited, particularly in indigenous goat breeds. By integrating these parameters within a repeated-measures design, the present study advances existing knowledge by clarifying the practical trade-offs between sampling techniques rather than merely confirming their utility.

### Diagnostic yield of swab stick method and pipette methods

Both sampling methods successfully retrieved vaginal epithelial cells; however, the swab stick method consistently yielded a significantly higher proportion of superficial cells, whereas the pipette method recovered more parabasal cells. This distinction is critical because superficial (cornified) cells are well-established cytological indicators of the follicular phase and oestrus in goats and other ruminants (17, 27, 28).

The difference in superficial cell yield was statistically significant (t = 2.34, *p* = 0.034) and of moderate magnitude (Cohen’s d = 0.58). The moderately large *t*-value indicates a real and practically important difference between methods, with the swab consistently producing higher superficial cell counts than the pipette, and the effect size confirms that this difference is biologically meaningful for oestrus detection.

Conversely, the higher recovery of parabasal cells by the pipette method was strongly significant (t = 4.06, *p* = 0.001) with a large effect size (d = 1.01), reflecting a robust methodological influence on epithelial cell composition. The large *t*-value indicates that parabasal counts were consistently higher in pipette samples than in swabs, and this difference is unlikely to be due to random sampling error. Although previous studies have reported epithelial cell fluctuations across the oestrous cycle (17, 29), the present findings refine this understanding by demonstrating that sampling technique itself substantially influences apparent cell-type proportions, even when animals are at the same physiological stage.

The higher parabasal cell recovery associated with the pipette method suggests deeper sampling of the vaginal epithelium, likely due to aspiration of vaginal fluid from mid-to-cranial regions, where the epithelium tends to contain younger, less keratinized cells. While this may be informative for assessing epithelial turnover, it is less advantageous when the primary objective is to accurately identify oestrus for breeding management. In contrast, the swab stick appears to preferentially collect superficial epithelial layers, enhancing its diagnostic sensitivity for heat detection. This methodological insight extends prior work by highlighting that cytological outcomes are not solely hormonally driven but are also shaped by pre-analytical sampling factors. Additionally, superficial (cornified) cells are characterized by absence of nucleus or the presence of a pyknotic nucleus, while intermediate cells possess a low nuclear-to-cytoplasmic ratio. In contrast, parabasal cells exhibit a high nuclear-to-cytoplasmic ratio (30). The higher parabasal cell yield observed with the pipette method could be attributed to this elevated nuclear-to-cytoplasmic ratio, which renders the cells relatively denser and consequently facilitates their suspension in the pipette’s collection fluid. Conversely, superficial cells are a nuclear and less dense, as previously reported (22). Superficial cells have been described as leaf-like (flat) because of their relatively low density (30). Thus, a considerable proportion of these lighter cells may have adhered to the inner wall of the pipette, explaining the lower yield obtained with pipette method compared to the swab stick method. The swab stick technique, which involves gently rolling the swab over the glass slide, likely allows for more efficient transfer of these lighter superficial cells. In other words, the proportion of less dense superficial cells was better represented in samples collected using the swab stick method, probably due to the nature of its smear preparation technique. This observation agrees with the report of Cora (21), who stated that pre-analytical sources of variation, including the collection technique, can affect cell density on vaginal cytology preparation.

### Smear clarity cellular integrity

Smear clarity and cellular integrity are central to reliable cytological interpretation. In this study, all pipette smears were free of cellular distortion, whereas the swab stick smears displayed mild to severe degrees of distortion. This aligns with the recommendation of Allison (31) on swab sampling technique, which states that epithelial cells should be transferred onto a glass slide by gently rolling the swab with minimal pressure to avoid rupturing cells and ensure adequate cell preservation. The pipette’s gentle aspiration technique, coupled with minimal contact pressure, likely preserved cellular morphology better, supporting its use in cytological studies requiring intact nuclear and cytoplasmic features. However, the higher proportion of heavy cellular debris in pipette smears, as observed in this study, may obscure epithelial cell visibility and hinder stage differentiation. This agrees with a published observation which reports that samples obtained using the pipette (lavage) method often contain mucus and exfoliated cellular debris, which dilute the epithelial cell population and reduce smear clarity, making cytological interpretation more difficult (32). This trade-off underscores a key methodological contribution of the study: better cellular integrity does not necessarily equate to better diagnostic clarity.

The abundance of debris in pipette samples may be linked to its sampling depth, where desquamation and mucosal secretions accumulate, whereas the swab primarily collects surface cells with less contamination. Although the pipette method produced smears with better cellular integrity, the swab stick method provided superior recovery of superficial epithelial cells, which are most critical for accurate estrus detection. Consequently, diagnostic clarity and biological relevance are more important than morphological preservation when selecting a routine sampling method for estrus monitoring. This nuanced comparison moves beyond binary assessments of “better” or “worse” methods and instead contextualizes technique selection based on diagnostic goals.

### Trauma and animal welfare

Animal welfare is an increasingly important consideration in reproductive monitoring, particularly when repeated sampling is required. In this study, the pipette method was associated with greater behavioral and cytological indicators of trauma, including prolonged bleating and a higher presence of blood cells on smears, whereas the swab stick method caused minimal discomfort. Our present findings contradict the observations of Ekambaram et al. (33), who reported that swab stick induced mechanical trauma and pseudo pregnancy in mice. The discrepancies in these studies could be species-specific because West African Dwarf goats are not commonly susceptible to pseudo pregnancy as seen in laboratory rodent (20).

Although trauma assessment was qualitative, the consistent pattern observed across animals suggests that swab sampling is less invasive and more suitable for serial oestrus monitoring. These findings contribute to existing literature by extending welfare-focused evaluations of vaginal cytology techniques to WAD goats, a breed for which such comparisons are scarce. Importantly, reduced discomfort during sampling may also minimize stress-related endocrine disruption, which can indirectly affect reproductive performance (34). Thus, from both ethical and physiological perspectives, the swab stick method appears preferable for routine reproductive monitoring. Nonetheless, it is imperative to acknowledge that the order of sample collection methods was not randomized, and an order effect cannot be completely excluded, as prior manipulation of the vaginal mucosa may have influenced trauma-related outcomes observed with the subsequent technique.

### Economic implications and time consumption

Economic feasibility and operational efficiency are critical for field application, especially in smallholder systems common in sub-Saharan Africa. While the pipette method was cheaper per unit and reusable, overall diagnostic cost-effectiveness favored the swab stick method due to its higher diagnostic yield, reduced trauma, and cleaner smears, which decrease the likelihood of repeat sampling. This integrated assessment demonstrates that lower unit cost does not necessarily translate to greater practical cost-effectiveness, a distinction often overlooked in methodological studies.

The swab stick method caused less trauma than the pipette, even though it required slightly more time to perform (15.91 ± 0.98 min vs. 12.48 ± 0.78 min), indicating that the lower trauma is attributable to the method itself rather than handling duration, and that handling time does not act as a confounding factor. While the pipette maintains better cellular integrity and is faster, potentially advantageous for large-scale or time-constrained applications, the swab consistently recovered more superficial epithelial cells and offered greater overall diagnostic effectiveness, which are essential for accurate oestrus detection. Taken together, these findings indicate that, in routine monitoring, the practical usefulness of the sample for accurate oestrus assessment is more important than simply preserving cell morphology, and they provide guidance for selecting sampling methods according to production and management priorities.

## Conclusion

The present findings indicate that the swab stick method is superior to the pipette method for routine vaginal cytology and oestrus detection in WAD goats. By yielding a higher proportion of superficial cells, minimizing animal trauma, and producing cleaner and more interpretable smears, the swab stick method enhances the accuracy of oestrus detection and supports improved reproductive management.

### Limitations of the study

We acknowledge that the small sample size (*n* = 4) limits statistical power, particularly affecting the descriptive statistics used in trauma assessment, and may reduce the generalizability of the findings, although this was partly mitigated by repeated sampling across multiple days. Sampling order was not randomized, which may have influenced trauma outcomes. The observation period was limited to a single synchronized cycle, and trauma assessment relied on qualitative indicators rather than objective stress biomarkers. Furthermore, findings are specific to WAD goats and may not be directly extrapolated to other breeds or production systems.

### Recommendation

- Future studies should validate these findings in WAD and other breeds of goat using larger sample sizes and integrate vaginal cytology with endocrine profiling, and stress biomarkers to provide a more comprehensive evaluation of both pipette and swab stick methods.
- Sampling order should be randomized to eliminate potential bias during trauma evaluation.
- To minimize cellular distortion associated with swab-based sampling, epithelial cells should be transferred onto glass slides by gently rolling the swab with minimal pressure, thereby reducing cell rupture and improving cellular preservation.

## Data Availability

The original contributions presented in the study are included in the article/supplementary material, further inquiries can be directed to the corresponding author.
